# Nanopore adaptive sampling for bacterial identification from periprosthetic joint replacement tissue

**DOI:** 10.1099/mgen.0.001507

**Published:** 2025-09-24

**Authors:** Teresa L. Street, Philip Bejon, Laura Leach, Sarah Oakley, Bernadette C. Young, Nicholas D. Sanderson

**Affiliations:** 1 NIHR Oxford Biomedical Research Centre, John Radcliffe Hospital, Oxford, UK; 2 Modernising Medical Microbiology, Nuffield Department of Medicine, University of Oxford, Oxford, UK; 3 Oxford University Hospitals NHS Trust, Oxford, UK

**Keywords:** adaptive sampling, clinical metagenomics, diagnostics, direct from sample sequencing, nanopore

## Abstract

Metagenomic approaches to the diagnosis of prosthetic joint infections promise more accurate and more rapid diagnosis. However, the high host DNA to bacterial DNA ratio is a challenge. Nanopore adaptive sampling (AS) can be used to preferentially sequence more of the infecting organism. Here, we evaluate AS using clinical samples from infected prosthetic joints to determine the absolute fold enrichment achieved. We found that AS achieved a range of 1.61- to 1.96-fold higher absolute fold enrichment for bacterial sequenced bases using AS over control pores. In this limited sample set, AS did not impact bacterial diagnosis overall but led to a modest increase in the bacterial sequence available without any obvious cost.

**Impact Statement**

Metagenomic approaches offer the possibility to rapidly detect the cause of an infection and to provide information on drug susceptibility. Implementing this technique is challenging because samples collected from patients contain high levels of human DNA which can obscure the detection of bacterial DNA. Reducing the amount of human DNA sequenced would allow easier detection of bacteria. This study assessed a sequencing protocol that rejects human DNA during the sequencing process known as adaptive sampling (AS), specifically as concerns samples from patients with joint infections. Our findings demonstrate that AS can increase bacterial sequencing efficiency. However, these modest improvements did not significantly enhance bacterial identification in our small sample set, although we did not detect additional costs associated with using AS. The study confirms the modest utility of AS in real-world clinical samples and extends the current literature by applying AS to joint infection. The implications of this method extend to clinical microbiology, where rapid and accurate pathogen detection can significantly impact patient outcomes.

## Data Summary

The authors confirm that all supporting data, code and protocols have been provided within the article, through supplementary data files or in publicly accessible repositories.

Nanopore sequencing fastq data are available in the ENA under project accession: PRJEB78709. Individual sample accession numbers are as follows: ERS23811994, ERS23811993, ERS23811992, ERS23811991, ERS23811990, ERS23811989, ERS23811988 and ERR14758464. Code used for analysis is available on GitHub at https://github.com/oxfordmmm/AS_analysis_scripts.

## Introduction

Direct-from-clinical-sample sequencing has been proposed as a method to diagnose infection more rapidly than culture-based methods and to diagnose non-culturable bacterial infection. However, clinical samples often contain high levels of host cells relative to bacterial cells, leading to the host DNA sequence swamping bacterial sequence on analysis [1]. Laboratory methods to reduce host nucleic acid contamination include differential lysis of host cells with saponin and degradation of the released DNA with nucleases. Although examples of success with this cheap and simple depletion method have been reported [2], some studies have found that this can cause bias in the bacterial composition of the sequenced sample [3] or adversely affect some species [4,5]. Other host depletion methods include leveraging the difference in methylation between human host and pathogen DNA [6] and osmotic shock of host cells followed by degradation of released DNA by propidium monoazide intercalation [7].

The Oxford Nanopore Technologies (ONT) sequencing platform can dynamically reject nucleic acid strands from specific pores during sequencing based on set criteria. This process, known as adaptive sampling (AS), has been used as a method to reduce the amount of host DNA sequenced in a variety of clinical samples and can improve the proportion of sequence data obtained from pathogens. For example, in respiratory samples, Gan *et al*. observed approximately a 3.6-fold increase in *Streptococcus pneumoniae* bases sequenced from sputum and bronchoalveolar lavage fluid (BALF) samples [8], and Lin *et al*. reported a 1.27- to 2.15-fold increase of human adenovirus data yield from human nasopharyngeal swabs [9]. Other sample types include vaginal swabs, where a 1.7-fold increase in bacterial sequencing depth was observed [10], and blood, where a 3- to 5-fold improvement for *Plasmodium falciparum* sequence was reported [11]. AS has also been applied to enrich sequence data for antibiotic resistance gene (ARG), with an eightfold increase in sequencing depth seen in BALF samples [12] and a twofold improvement in ARG sequencing depth from soil samples for environmental surveillance [13]. On the other hand, others have found that AS provided little or no sequence enrichment. Lin *et al*. observed no enrichment of pathogen sequence data for *Chlamydia psittaci* from a BALF sample [14], although the authors acknowledge that the single sample used was likely partially degraded due to extended storage prior to extraction.

Joint replacement is a highly cost-effective procedure to treat pain and restore mobility but is complicated by infection in 1–2% of cases [15,16]. The infecting bacteria may be present in low numbers and may be difficult to distinguish from contaminants. To address this, traditional microbiological methods are applied to multiple tissue samples taken at the time of surgery, and these are cultured for extended incubation periods [17,18]. Tissue samples by definition include a large proportion of host DNA. Clinical metagenomic sequencing has been tested for bone and joint infection by ourselves and others [4,19, 20], with studies identifying high levels of contaminating host DNA as a hurdle to robust and reliable detection of infecting species. Previous attempts have been made using saponin to deplete host DNA in prosthetic joint infection (PJI) samples prior to sequencing, but these methods were not completely pathogen agnostic [4]. To our knowledge, nanopore sequencing with AS has not been tested for samples originating from bone and joint infections.

AS can be applied using two different strategies, with enriching for a specific target (enrich) such as a known pathogen reference genome or depleting a specific target (deplete) such as the human reference genome. The enrich strategy is theoretically more efficient in rejecting all reads that do not match the target but is not applicable to a metagenomic approach on clinical samples where knowledge of the target is lacking. Here, we evaluate AS for its usefulness as a host depletion tool in clinical metagenomics. Using culture-positive periprosthetic tissue (PPT) samples, we test AS using the depletion method for contaminating host DNA removal.

## Methods

### Sample preparation and routine microbiology

PPT samples were collected intraoperatively during revision arthroplasty surgery at the Nuffield Orthopaedic Centre of Oxford University Hospitals, UK, and obtained for this evaluation following routine diagnostic workup (NHS research ethics committee reference 17/LO/1420). Seven samples were selected at random from amongst culture-positive samples identified by routine microbiological assessment of PPT during two separate weeks in March and July 2024. Routine processing of PPT samples by the microbiology laboratory was as follows: Bactec bottles were inoculated with 0.5 ml of an inoculum generated by vortexing each tissue sample in 3 ml of 0.9% saline with sterile Ballotini balls for 15 s. Bottles were incubated under aerobic (Plus Aerobic/F culture vials) and anaerobic (Lytic/10 Anaerobic/F culture vials) conditions in a BD Bactec FX system (BD Biosciences) for up to 10 days. Any bottles that flagged positive were subcultured onto agar plates, and all cultured micro-organisms were identified by MALDI-TOF MS on a Microflex LT using Biotyper, version 3.1 (Bruker Daltonics).

#### DNA extraction

DNA was extracted from the saline inoculum, described above after vortexing with Ballotini balls. One millilitre was passed through a 5 µm syringe filter to remove any tissue debris. DNA was extracted from 500 µl of the filtered sample using the UCP Pathogen Mini kit (Qiagen) as per the manufacturer’s instructions, following mechanical lysis on a FastPrep-24 (MP Biomedicals), and eluted in 50 µl. Prior to preparation for sequencing, DNA was purified using 1.8× AMPure XP beads (Beckman Coulter), eluted in 20 µl molecular biology-grade water and quantified on a Qubit 4.0 fluorimeter with the Quant-iT dsDNA HS Assay kit (Life Technologies). A saline-only sample (i.e. with no PPT) was also processed in parallel as a negative control.

#### Library preparation and sequencing

Sample 1 was prepared for sequencing using the Ligation kit (SQK-LSK110, ONT) using 1 µg DNA as per the manufacturer’s instructions. Nineteen femtomoles were loaded onto a single washed v9.4.1 flow cell (with 887 pores available at the start of sequencing) and sequenced for 72 h on a GridION MK1 with basecalling performed after sequencing using Guppy 6.3.4 and dna_r9.4.1_450bps_hac.cfg. AS was performed with depletion on pore numbers 1–256 using the human reference genome (GCF_000001405.40_GRCh38.p14_genomic), with pores 257–512 used as no-depletion controls – hereafter referred to as non-AS.

Samples 2–7 did not generate sufficient DNA for preparation by ligation sequencing (400 ng requirement for native barcoding ligation), so were prepared for sequencing using the Rapid PCR Barcoding kit (SQK-RPB114.24, ONT). Five nanograms of DNA, or, where this was not possible, 3 µl total volume, were used as input per sample according to the manufacturer’s instructions and including the negative control. Post-PCR, 133 ng of each sample was pooled and cleaned, and 50 fmol of the final pooled library was loaded onto a v10.4.1 flow cell and sequenced for 72 h on a GridION MK1 with AS performed as described above. Basecalling for these samples was carried out in real time on the GridION with Dorado and the dna_r10.4.1_e8.2_400bps_hac@v4.3.0 model.

### Bioinformatics analysis

Basecalled fastq files were classified with Kraken2 [21] (v2.1.3) using a database containing human, bacterial, archaeal and viral genomes (k2_standard_20240112, available at https://genome-idx.s3.amazonaws.com/kraken/k2_standard_20240605.tar.gz). Each read was assigned as human, target species or other, based on the highest taxonomic classification for the read to calculate yield improvements. Reads classified at the species level and above were mapped to a reference genome, and mapping statistics were generated where there were greater than ten reads classified to a single species. Reference genomes were chosen automatically from those designated ‘reference_genome’ in NCBI ‘refseq_category’. Where a reference genome was not available for any given species, the first ‘representative_genome’ available was used instead using the getRef.py script in the GitHub repository. Median read lengths were calculated for each sample using a subsample of up to 5,000 reads. Barcode crossover effects may arise from imperfect demultiplexing and read misclassification, leading to apparent contamination where a species in another sample in the same sequencing run yields very high coverage. This was addressed by applying a heuristic exclusion threshold: any species with a genome coverage breadth of <5% was not considered to be a true species. Analysis scripts are available at https://github.com/oxfordmmm/AS_analysis_scripts. Basecalled fastq files were also classified with Sylph using the default settings [22] for comparison with Kraken2 and mapping. The GTDB R220 database was used (gtdb-r220-c200-dbv1.syldb), and the effective coverage was reported. To calculate normalized bases, the number of unique active channel-pore combinations at the beginning of the sequencing run per AS and non-AS group was used as the denominator.

## Results

### PPT samples

Seven culture-positive PPT samples from seven individual patients were included in this evaluation, with one sequenced individually on a single flow cell and six sequenced with a single negative control as a multiplex on a further flow cell. All samples were culture-positive as follows: sample 1, *Enterococcus faecalis*, *Escherichia coli*, *Staphylococcus aureus*; sample 2, *E. coli*; sample 3, *E. faecalis*, *Proteus mirabilis*, *Streptococcus constellatus*; sample 4, *Enterobacter cloacae* complex, *A. baumannii*; sample 5, *E. coli*, coagulase-negative *Staphylococcus*; sample 6, *S. aureus*; and sample 7, *S. aureus*, coagulase-negative *Staphylococcus*.

### Sequencing results

Three samples (samples 5, 6 and 7) generated very low numbers of bacterial reads (i.e. <600), compared with 602 bacterial reads in the negative control; hence, these samples were regarded as negative for the purpose of this evaluation and were not included in further analysis of AS.

**Fig. 1.**
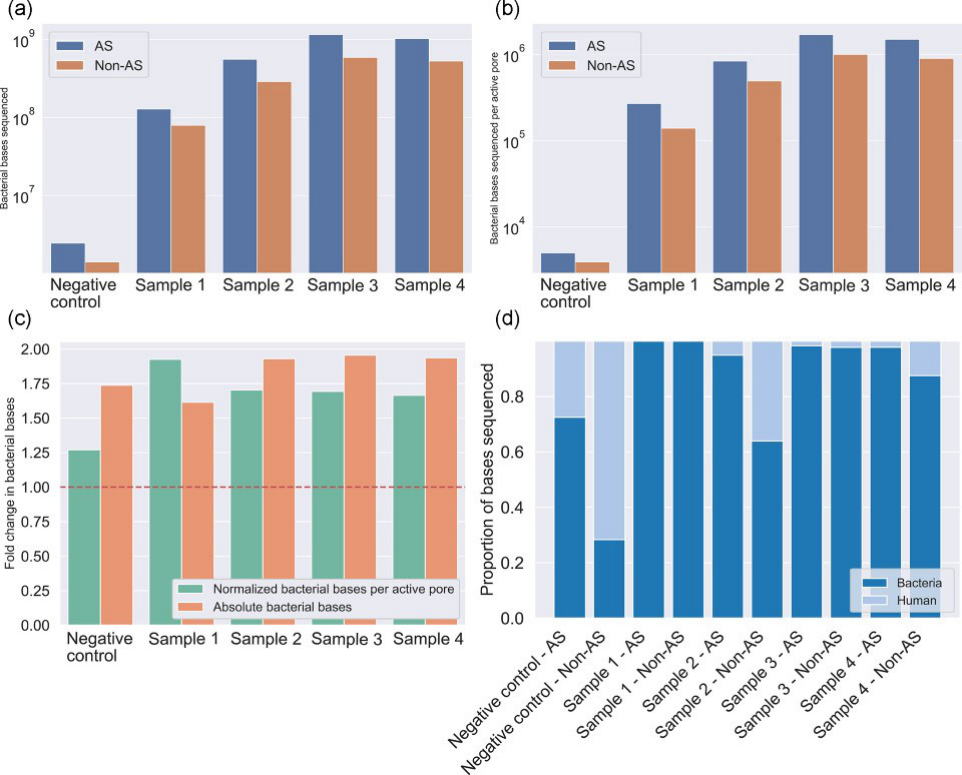
Bar charts showing comparison of bacterial and human bases sequenced from the AS group compared to the non-AS group. (a) absolute bacterial bases; (b) bacterial bases normalized by active pores at the start of the run; (c) fold change in bacterial bases with absolute bacterial bases (orange) and bacterial bases normalized by active pores (green); the red dotted line indicates 1× fold change where no difference would be observed between non-AS and AS; (d) proportion of bacterial and human bases for each sample.

Sample 1 was sequenced on a single flow and, as expected, generated more data per sample (6874.7 Mb) than the other samples after multiplexed sequencing [median 1,117.9 Mb; interquartile range (IQR) 940.2–1,414.5 Mb] (Table 1). AS caused a substantial reduction in human read lengths compared to the non-AS group (Table 2) due to rejection of these reads on mapping to the human reference genome. Median lengths of range 2.7–5.1 kb in the non-AS group reduced to 548–699 bp in the majority of the AS group. The exception was sample 3, where the median read length reduced from 5 kb to only 4.2 kb. However, there were only 2,652 human reads for sample 3 in the AS group, representing only 0.67% of the total reads generated with AS for this sample. Overall, a reduction in observed median human read length, indicative of rejection of human reads by the pores, enabled more bacterial bases to be sequenced in the AS group. Table S1, available in the online Supplementary Material, shows the number of reads unblocked (rejected) and their subsequent classification by Kraken2 in the AS group for each sample. The number of bacterial bases increased from a median (IQR) of 288.7 Mb (79.4–529.0 Mb) in the non-AS group to 557.1 Mb (128.1–1,024.5 Mb) with AS (Fig. 1a).

**Table 1.** Run metrics for PPT samples successfully sequenced. Control (non-AS) and AS group read numbers and megabases (1 million bases) shown, with proportions as a percentage of the total reads for each group given in brackets. Absolute enrichment is the fold increase in bases for bacteria between non-AS and AS groups

		Absolute reads (proportion as %)						Absolute megabases (proportion as %)						
Sample name	Multiplexed	Bacteria non-AS	Bacteria AS	Human non-AS	Human AS	Other non-AS	Other AS	Bacteria non-AS	Bacteria AS	Human non-AS	Human AS	Other non-AS	Other AS	Absolute enrichment
Sample 1	No	19,321	31,649	1,166,387	1,950,380	277,300	434,284	79.13	127.64	4,100.59	1,146.81	803.35	617.14	1.61
		(1.32)	(1.31)	(79.73)	(80.72)	(18.95)	(17.97)	(1.59)	(6.75)	(82.29)	(60.63)	(16.12)	(32.63)	
Sample 2	Yes	92,598	179,808	55,546	111,524	3,276	6,438	280.83	541.55	162.8	101.41	10.55	20.75	1.93
		(61.15)	(60.38)	(36.68)	(37.45)	(2.16)	(2.16)	(61.83)	(81.59)	(35.84)	(15.28)	(2.23)	(3.13)	
Sample 3	Yes	197,896	389,633	1,542	2,652	1,096	2,195	588.35	1,150.80	13.74	20.84	2.58	5.14	1.96
		(98.68)	(98.77)	(0.77)	(0.67)	(0.55)	(0.56)	(97.30)	(97.79)	(2.27)	(1.77)	(0.43)	(0.44)	
Sample 4	Yes	168,747	329,714	24,577	47,611	2,013	4,002	526.62	1,020.02	75.6	53.31	5.37	10.51	1.94
		(86.39)	(86.46)	(12.58)	(12.49)	(1.03)	(1.05)	(86.67)	(94.11)	(12.44)	(4.92)	(0.88)	(0.97)	
Negative control	Yes	213	389	674	1,415	270	532	1.09	1.83	3.56	1.77	0.79	1.52	1.69
		(18.41)	(16.65)	(58.25)	(60.57)	(23.34)	(22.77)	(20.04)	(35.74)	(65.44)	(34.57)	(14.52)	(29.69)	

**Table 2.** Read length median (IQR) for control (non-AS) and AS groups, classified by Kraken2 as bacteria, human or other and generated using a subsample of up to 5,000 reads per taxonomic group

	Bacteria		Human		Other	
Sample name	Non-AS	AS	Non-AS	AS	Non-AS	AS
Sample 1	3,570	3,575	3,237	548	2,611	701
	(3,029, 4,828)	(3,156, 4,810)	(1,981, 4,750)	(493, 607)	(876, 4,059)	(531, 1,837)
Sample 2	2,984	2,981	2,795	699	3,217	3,204
	(2,228, 3,787)	(2,254, 3,730)	(2,325, 3,300)	(609, 812)	(2,631, 3,876)	(2,596, 3,891)
Sample 3	2,833	2,951	5,027	4,154	2,511	2,449
	(2,330, 3,436)	(2,495, 3,503)	(2,796, 9,842)	(926, 8,578)	(1,773, 3,006)	(1,682, 3,034)
Sample 4	3,047	3,061	2,704	692	2,769	2,693
	(2,508, 3,630)	(2,514, 3,642)	(2,259, 3,250)	(595, 821)	(2,151, 3,289)	(2,079, 3,230)
Negative control	5,136	4,788	5,100	683	4,251	4,243
	(3,024, 6,769)	(3,083, 6,483)	(4,049, 5,662)	(598, 798)	(167, 4,365)	(163, 4,370)

When accounting for the number of active pores per AS or non-AS group, we also observed an increase in the number of bacterial bases with AS, increasing from a median (IQR) of 491.7 Kb (139.5–898.1 Kb) in the non-AS group to 837.7 Kb (268.6–1,495.7 Kb) with AS (Fig. 1b).

Comparing bacterial bases generated between AS and non-AS groups shows a positive fold enrichment across all samples when AS is applied, with a median (IQR) fold improvement of 1.83× (1.74–1.94×) for absolute bacterial bases and 1.65× (1.67–1.70×) for normalized bacterial bases (Fig. 1c, d). Findings from read mapping were confirmed by analysis with a k-mer-based classifier (Sylph), which similarly showed a median (IQR) yield improvement of 1.71× (1.28×–1.97×) for effective coverage (Table S2).

Within sample reads from multiplexed samples, we detected low levels of bacterial reads mapping to species present in abundance in other samples on the run [e.g. *Morganella morganii* detected in high read numbers in sample 3 and lower in sample 2 (Table 2)]. We hypothesize that this is a signal of demultiplexing error or barcode crossover. This is supported by the observation that the demultiplexing performed on the sequencing device also allocated sequence reads to barcodes not used in this experiment (Fig. S1). This remains a challenge in multiplex sequencing. We applied a heuristic exclusion threshold for species detection, as outlined in the bioinformatic methods, to reduce false-positive detection from barcode crossover.

### Bacterial species identified

The number of bacterial bases classified to the species level and aligned to reference genomes shows that AS increased both the yield and genome coverage breadth for the species identified by sequencing for each sample (Tables 3 and S3).

**Table 3.** Number of mapped sequence bases, reads and genome coverage breadth and depth split by control (non-AS) and AS groups. Top 5 most abundant species with AS by the number of bases per sample included. Species meeting exclusion thresholds of <5% genome coverage breadth not included. Additionally, any species identified in the sample culture are reported

	Mapped bases, bp (mapped reads)		Genome coverage breadth*, %		Genome coverage depth†, fold	
Species	Non-AS	AS	Non-AS	AS	Non-AS	AS
Negative control						
None						
Sample 1						
*E. faecalis*‡	50,799,756 (12,689)	81,613,199 (20,527)	91.93	92.00	19.69	31.61
*E. coli*‡	14,245,777 (5,263)	24,448,342 (8,922)	0.14	0.12	1,883.62	3,617.69
*S. aureus*‡	39,597 (9)	137,715 (27)	1.40	4.64	1.00	1.05
Sample 2						
*E. coli*‡	113,588,135 (43,016)	220,371,387 (83,524)	71.34	71.54	28.96	56.02
*Escherichia albertii*	345,063 (146)	648,291 (274)	3.13	5.91	2.36	2.34
*Enterobacter hormaechei*	418,243 (162)	641,093 (241)	9.10	13.12	1.09	1.16
*M. morganii*	369,560 (141)	566,543 (209)	8.94	12.94	1.06	1.13
Sample 3						
*M. morganii*	450,678,041 (171,403)	881,583,379 (337,175)	93.35	93.35	124.11	242.79
*P. mirabilis*‡	34,580,859 (13,6260	67,693,728 (26,814)	91.99	92.69	9.25	17.97
*E. hormaechei*	345,093 (120)	706,131 (242)	7.26	14.64	1.13	1.15
*Porphyromonas asaccharolytica*	400,823 (177)	656,033 (316)	15.88	23.39	1.15	1.28
*Campylobacter ureolyticus*	180,341 (76)	310,566 (139)	1.07	14.51	9.30	1.18
*E. faecalis*§	65,387 (27)	294,621 (115)	2.08	9.41	1.12	1.12
Sample 4						
*E. hormaechei*‡	273,777,858 (99,876)	529,556,235 (193,837)	87.06	87.26	74.91	144.57
*Enterobacter* sp. *BIDMC100*‡	11,568,088 (3,918)	22,584,779 (7,719)	20.46	27.42	12.44	18.12
*E. cloacae*‡	7,303,047 (2,837)	13,862,857 (5,472)	43.19	53.52	3.46	5.30
*E. cloacae* complex sp*.*‡	6,309,685 (2,343)	12,248,561 (4,634)	43.44	54.02	3.04	4.74
*Enterobacter roggenkampii*‡	5,717,882 (2,391)	11,137,693 (4,647)	34.57	45.95	3.48	5.11
*A. baumannii*§	472,685 (181)	853,225 (311)	10.15	17.91	1.17	1.20

*Percentage of the reference genomes sequenced to at least 1× depth classified by Kraken2.

†Non-zero average sequencing coverage depth of the sequenced reference genomes classified by Kraken2; positions with no coverage are not included in the average calculation.

‡Species identified by culture for the corresponding sample.

§Species detected by culture for the corresponding sample but not in the top 5 most abundant species with AS.

The number of species-level bases increased from a median (IQR) of 114.4 Mb (65–298.9 Mb) in the non-AS group to 221.7 Mb (106.2–577.9 Mb) with AS.

The negative control sample contained reads mapping to 14 species, but these were for relatively low numbers of bases, and all were removed by our exclusion thresholds (Table S3); these reads likely represent kitome or barcode crossover due to the large numbers of reads corresponding to some species seen in other samples within the multiplex and are therefore not considered to be genuine.

Sample 1 was culture-positive for *E. faecalis*, *E. coli* and *S. aureus*, and all three species were identified by sequencing (Tables 3 and S3). The low read numbers classified as *S. aureus* would have been interpreted as non-significant in the absence of the culture result, given the low breadth of genome covered. Similarly, *E. coli*, which was detected with very low breadth despite very high depth of genome coverage (Table 3), was also filtered out by our genome breadth exclusion threshold as contamination. Detailed analysis of these reads maps them to two plasmids which are part of the accessory genome of the *E. coli* reference strain chosen (Fig. S2), indicative of ‘kitome’.

In the other polymicrobial samples, *S. constellatus* (sample 3) was detected below our mapping quality control threshold in both the AS and non-AS groups; *Streptococcus anginosus* was detected and mapped in both groups, and this could represent bioinformatic misclassification due to low base numbers and similarity between species (both being members of the *S. anginosus* group) (Table S3). The largest proportion of species-level reads in sample 3 were classified as *M. morganii* (Table 3), a species not detected by routine culture in the sample chosen for this evaluation but cultured in additional PPT samples collected concurrently from the same patient. Two additional species, *P. asaccharolytica* and *C. ureolyticus*, were also detected in sample 3, with AS improving the genome coverage breadth of both. *E. hormaechei* detection in this sample is likely due to barcode crossover given the large number of reads classified to this species present in sample 4.

Sample 4 was dominated by *Enterobacter* reads classified as multiple separate species (Table 3), but likely representing a single species with artefactual bioinformatic misclassification. As a result of the overwhelming number of *Enterobacter* bases in both the non-AS and AS groups, *A. baumannii*, the other species detected by culture in this sample, was detected outside the top 5 most abundant species classified. AS did, however, improve the number of bases classified and the breadth of genome coverage of this species.

In the case of a sample monomicrobial by culture, AS almost doubled the number of bases classified as *E. coli* in sample 2, which was culture-positive for this species (Table 3). AS improved genome coverage depth but not breadth, which was likely at the maximum coverage breadth for the automatically selected reference given the diversity of *E. coli* pangenomes. In addition to *E. coli*, bases also mapped to an additional *Escherichia* species, suggestive of bioinformatic misclassification. Barcode crossover likely explains the apparent detection of *E. hormaechei* and *M. morganii*.

## Discussion

AS, when applied to bone and joint infection, led to a modest reduction in the read lengths of human DNA without affecting bacterial DNA reads. By rejecting human reads, the aim of AS is to make sequencing pores available more frequently to sequence the organism DNA of interest. All four PPT samples with sufficient bacterial reads for analysis generated a 1.6- to 2-fold enrichment for bacterial sequence with AS compared to the non-AS control group. This is consistent with previous reports [8,10, 12].

We generated low numbers of bacterial reads for three of seven samples. We propose that this was due to a combination of factors, including the overwhelming amount of human DNA, inefficient extraction of bacterial DNA with the protocol used and the presence of species at low bacterial loads. *S. aureus* was only detected in sample 1 at limited genome breadth and depth and would have been interpreted as non-significant without the benefit of culture results. In addition to inefficient DNA extraction from this Gram-positive organism, there was also competition from the large number of *Enterococcus* bases sequenced in this sample. *S. constellatus* was detected at very low levels in sample 3, and this also likely represents inefficient DNA extraction given that all 5 PPT samples from this patient were culture-positive for this *Streptococcus*. Despite AS improving genome coverage breadth, *Acinetobacter baumannii* was only detected at low levels in sample 4, which we propose was due to the very high numbers of *Enterobacter* species dominating the bacterial reads for this sample.

Sample 3 was a polymicrobial sample with 6 species cultured from the 5 PPT samples collected. The very large proportion of *M. morganii* reads detected in this sample, and the fact that two of four additional PPT samples collected from this patient cultured *M. morganii*, suggests true infection, despite the sample tested here being culture-negative for this species. Additionally, the large number of *M. morganii* DNA fragments likely outcompeted DNA from other species during sequencing, despite this sample having a relatively low fraction of human reads. In sample 4, it is likely that the mapping to multiple species reflects a single *Enterobacter* infection, with related species competing for read mapping in the workflow used.

Thresholds for species detection in metagenomic sequencing of bone and joint infection will need careful evaluation, and robust thresholds will be required to assess potentially low-bacterial-load samples. This will be important for addressing potential false-positive and false-negative results. *S. aureus* was detected just below a heuristic threshold of 5% coverage in sample 1 and was also found in culture of that sample, as well as in 1 of 4 other PPT samples from the same patient. Previous work on the microbiology of PJI suggests that a single culture-positive PPT sample is poorly predictive of genuine infection, and the highest predictive value comes from finding an organism in three or more samples [17]. Two additional species detected in sample 3, *P. asaccharolytica* and *C. ureolyticus*, met heuristic thresholds for this study but are unexpected species in bone and joint infection, and it is unclear if they were significant species in this context.

Sample 1 was culture-positive for *E. coli*, and although this species was detected by sequencing, it is more likely to represent kit contamination, or ‘kitome’ [23,24], given the very limited genome breadth observed (despite the large number of bases and depth of coverage) and the fact that these reads map to plasmids in the accessory genome of the *E. coli* reference strain used. In this sample, we see evidence that AS will amplify both true pathogens and contaminant nucleic acid.

Barcode crossover, as observed here and by others [25], resulting from imperfect demultiplexing and read misclassification, impacts species detection. Validation of the entire workflow from DNA extraction to sequence data interpretation will be necessary to interpret true positives from false positives arising due to barcode carryover and ‘kitome’ contamination. This is particularly important in the case of bone and joint infection, where samples can often have low bacterial loads.

Limitations of this evaluation include splitting the flow cell by pore number into non-AS and AS groups. A further study with greater sample numbers and controls run on separate flow cells would assess the impact of this, although variation in available pore numbers on different flow cells would also influence the interpretation of results here. Additionally, the bacterial reference genomes used were not masked for any shared sequence identity between host and target organism, and this may have reduced the specificity of the AS host read classifications resulting in erroneously unblocked reads [26]. Finally, as seen in *E. coli* culture-positive sample 2, automatic reference selection using NCBI reference genomes per species is a limitation. Highly diverse species may not achieve high genome coverage breadth due to imperfect choice of reference genome.

Multiplexing samples limits the cost of metagenomic sequencing but does, however, limit the data output for each individual sample. This impacts detection of low-abundance species, and the possibility of barcode crossover with multiplexing may further complicate interpretation. Metagenomic sequencing can achieve greater sensitivity with greater sequencing depth, but this is also more costly. All diagnostic tests face a trade-off between sensitivity and specificity. Prioritizing the specificity of findings delivers more actionable results and may be more cost-effective, although clinical trials and health economic reviews would be needed to verify this.

AS can increase the proportion of bacterial bases sequenced from metagenomic extractions. Whilst the fold enrichment observed here and by others does not offer order-of-magnitude improvements in bacterial sequence yield, it is a simple and useful addition to a metagenomic diagnostic workflow, and there does not appear to be any harm from using AS. The additional pathogen sequence data generated could theoretically contribute to the identification of low bacterial load species and enhance antimicrobial resistance determinant detection, but we were unable to demonstrate this with the limited number of samples in this study at the modest fold enrichment observed.

## Supplementary material

10.1099/mgen.0.001507 Uncited Supplementary Material 1.

10.1099/mgen.0.001507 Uncited Supplementary Material 2.

## Abbreviations

ARG: antibiotic resistance gene
AS: adaptive sampling
BALF: bronchoalveolar lavage fluid
ONT: Oxford Nanopore Technologies
PJI: prosthetic joint infection
PPT: periprosthetic tissue
