# Supplementary material for: Nanopore adaptive sampling for bacterial identification from periprosthetic joint replacement tissue
Source: Microb Genom. 2025 Sep 24;11(9):001507. doi: 10.1099/mgen.0.001507 (PMC13293318; doi:10.1099/mgen.0.001507)
Supplement: Uncited Supplementary Material 1. [file mgen-11-01507-s001.pdf]

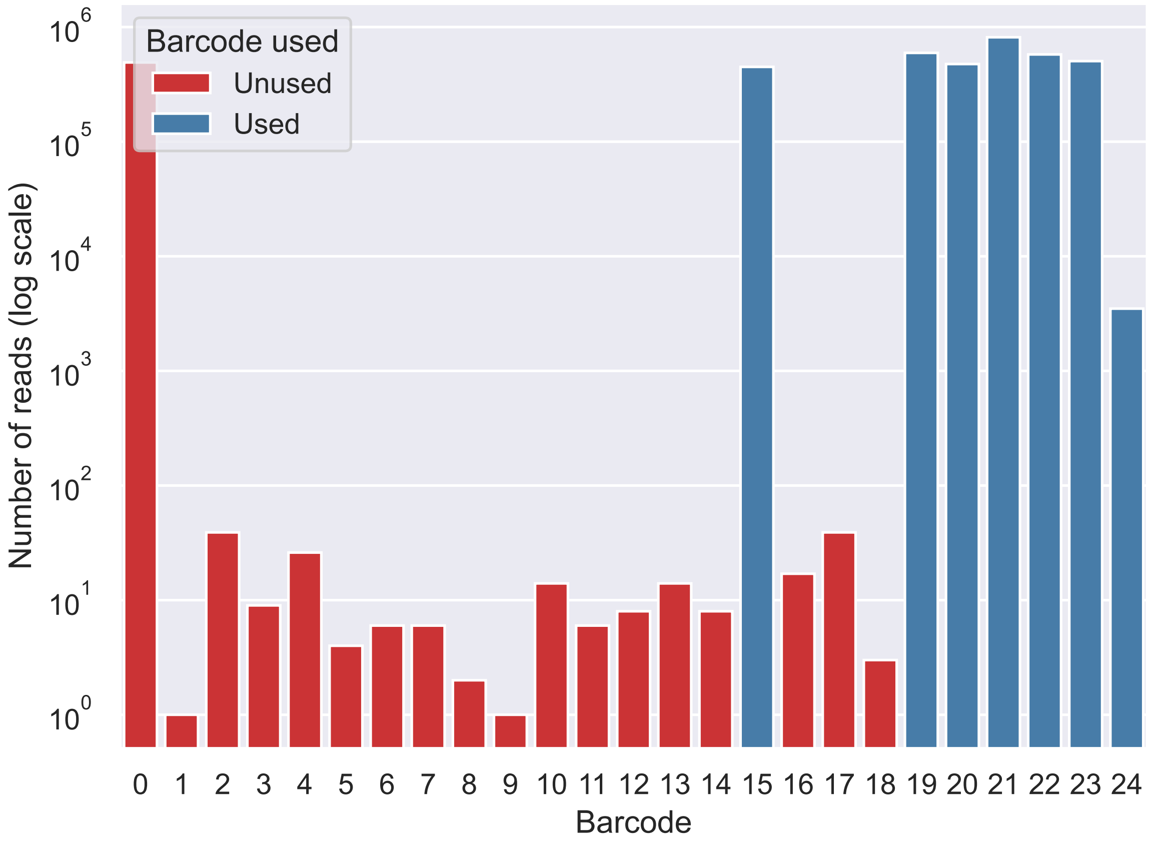

Figure S1. Reads assigned to barcodes by Dorado during demultiplexing on the GridION. Blue represents barcodes used in the multiplexed sequencing run. Red represents barcodes not used in the sequencing run. Barcode 0 represents unassigned reads.

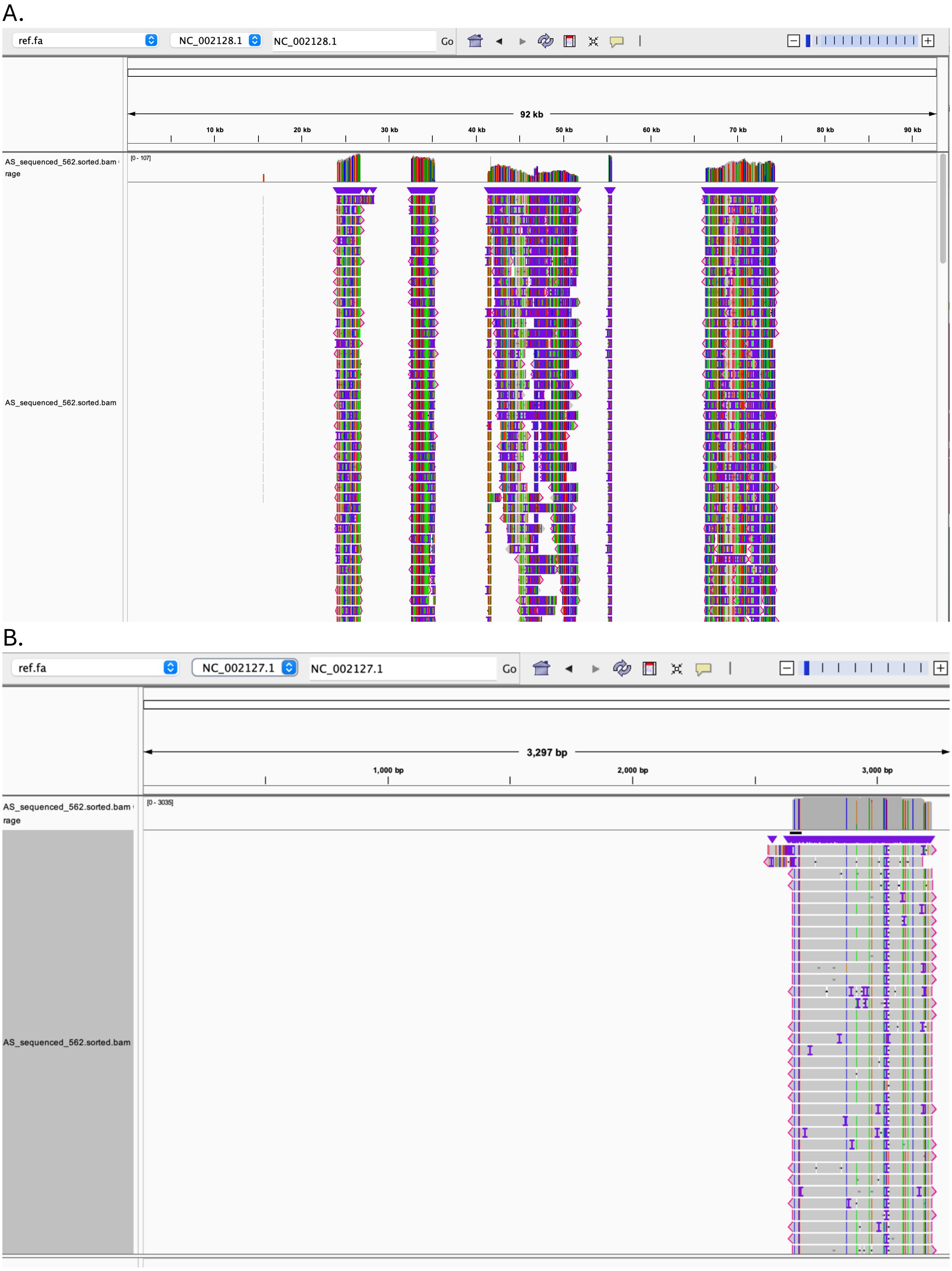

Figure S2. Mapping data for reads classified as *E. coli* from sample 1. A and B highlight the regions on two plasmids from the *E. coli* accessory genome where the reads map.
